# Impact of various dietary lipids on amelioration of biomarkers linked to metabolic syndrome in both healthy and diabetic Wistar rats

**DOI:** 10.1186/s40795-024-00881-7

**Published:** 2024-05-16

**Authors:** Weaam I. Abudigin, Adnan Bajaber, Pandurangan Subash-Babu

**Affiliations:** Department of Food Science and Nutrition, College of Food & Agriculture Sciences, King Saud University, P.O. Box 22452, Riyadh, 11459 Saudi Arabia

**Keywords:** Dietary lipids, Saturated fats, Oxidative stress, Inflammation, Cardiovascular diseases

## Abstract

**Background:**

The present study was designed to investigate the influence of different dietary lipids (sheep’s fat, olive oil, coconut oil, and corn oil) on specific biomarkers associated with metabolic syndrome in both healthy and diabetic rats.

**Methods:**

The study designed for 45 days, utilized a male diabetic wistar rat (body weight, 180–220 g) model induced by streptozotocin (45 mg/kg bw). The rats were divided into two sections: five non-diabetic and five diabetic groups, each containing six rats. The first group in each section serving as the control, received a standard diet. Both non-diabetic or diabetic groups, were provided with a standard diet enriched with 15% sheep fat, 15% coconut oil, 15% olive oil, and 15% corn oil, respectively for a duration of 45 days.

**Results:**

Post-supplementation, both healthy and diabetic control rats exhibited a higher food intake compared to rats supplemented with lipid diet; notably food intake was higher in diabetic control than healthy control. However, rats fed with coconut oil, olive oil and sheep fat showed weight gain at the end of the experiment, in both healthy and diabetic groups. Coconut oil supplementation significantly (*p* ≤ 0.05) increased HDL-C and total cholesterol level in diabetic groups compared to healthy group, it was confirmed by an increased PPAR-α and ABCA-1 protein level. Olive oil significantly decreased triglyceride, total cholesterol, and LDL-C levels in diabetic rats when compared to sheep fat or coconut oil. Corn oil significantly decreased fasting glucose, total cholesterol and LDL-C levels compared to all other groups. Corn and olive oil supplemented normal groups, found with significant increase in hepatic glucose-lipid oxidative metabolism associated protein, like FGF-21, MSH, ABCA-1, PPAR-γ and decreased lipogenesis proteins like, SREBP and PPAR-α levels. In contrast, sheep grease and coconut oil increased SREBP and PPAR-α expression in both normal and diabetic groups. Most notably, normal and diabetic groups pretreated with sheep grease resulted in increased inflammatory (MCP-1, IL-1β, TLR-4, TNF-α), and oxidative stress markers (LPO, GSH, GPx, SOD and CAT) linked with metabolic complications.

**Conclusion:**

The combination or alternative use of olive oil and corn oil in daily diet may play a significant role in preventing proinflammatory condition associated with insulin resistance and cardiovascular diseases.

## Background

Obesity is characterized by the excess of body fat and it is distinct from insulin resistance. It may not be considered a detrimental condition as long as the fat is transported by the lipoproteins and stored in healthy fat cells that respond to insulin. In contrast, insulin resistance conditions the cells are poorly or not responding appropriately to circulating insulin [1]. Alterations in lipid homeostasis in live and insulin sensitive tissues – are pivotal in the onset of decreased insulin sensitivity, impaired glucose absorption, hyperglycemia and type 2 diabetes mellitus (T2D) [2]. Dietary modifications can potentially mitigate or reverse these alterations by influencing fatty acid homeostasis [3]. Apart from an increased fat mass, dysfunctional adipose tissue, characterized by an altered lipid storage capacity and adipokine secretion, can lead to hyperlipidemia, systemic inflammation and excessive lipid flow and storage in non-adipose tissues. These dysfunctions collectively contribute to the development of inflammation, insulin resistance, T2D and atherogenesis [4].

Energy intake and diet combinations, including dietary fats, carbohydrate and fibers, can exert a considerable influence on various aspects of fatty acid metabolism in peripheral and insulin sensitive tissues [5]. Stinkens et al. [6] noted that basal lipoprotein lipase (LPL) activity tends to rise in obese individuals. However, during hyperinsulinemia, the suppression of the uptake of liberated free fatty acids (lipid spillover) from LPL-mediated triglyceride hydrolysis in adipose tissue is less effective compared to lean, healthy individuals. Furthermore, impaired triglyceride clearance across adipose tissue is observed in obesity, insulin resistance, and type 2 diabetes (T2D) due to reduced insulin-mediated stimulation of LPL activity. This suggests a less efficient removal of dietary lipids by adipose tissue in these conditions [7]. McQuaid et al. [8] have demonstrated that the relative quantity of dietary fat stored in adipose tissue after the intake of subsequent meals was significantly reduced in abdominally obese vs. lean individuals.

The progressive globalization of the food industry has enabled the widespread availability with an affordable cost, of a diverse range of packed food and pastries, often with high calories. Majority of these food are rich in saturated fatty acids (SFA) and hydrogenated or trans fatty acids (TFA), which are likely represent the prominent risk factors for healthy living together with regular diet [9]. Consequently, the consumption of excessive dietary fats or nutrients stimulates adipocyte hypertrophy and obesity after excessive LPL mediated TAG hydrolysis and low in relative quantity of dietary fat storage [5]. During the progression of obesity, dynamic shifts occur within the immune cell populations in adipose tissue. This transition involves a change from an anti-inflammatory state to pro-inflammatory conditions, leading to a progression of low-grade inflammation. These alterations impact insulin sensitivity through various mechanisms [10]. While various molecular factors can contribute to insulin resistance either directly or indirectly from enhanced inflammation progress [11]. It holds significance because all dietary nutrients inherently possess inflammatory potential, given that their metabolism into other biological materials or conversion to energy can elicit molecular responses leading to increased inflammation [12]. In the presence of elevated inflammation, the capacity of insulin to regulate metabolism is compromised, which resulted in the pathogenesis of insulin resistance and cardiovascular disorders [13].

In the present research, we examine the effects of different dietary lipid supplementation along with standard diet, on changes in body weight, plasma glucose levels, lipid profile and key metabolic indicators such as lipid profile, oxidative stress and inflammatory markers. The experimental design includes the supplementation of distinct lipid sources to the healthy as well as diabetic rats, to elucidate their differential effects on the alterations in inflammatory markers associated with metabolic syndrome progression. The research findings may contribute valuable insights into the pronounced relationship between dietary lipids and metabolic health, offering potential implications for dietary interventions in individuals with diabetes and metabolic syndrome.

## Materials and methods

### Chemicals

Streptozotocin (STZ) was purchased from Sigma chemical co., St. Louis, MO, USA. All the blood and lipid profile analysis have been carried out using kits methods and related chemicals were of molecular biology grade purchased from Sigma chemical Co., MO, USA. All spectrophotometric measurements were carried out using UV2010 Spectrophotometer (Hitachi, Germany).

### Animals

Adult male albino Wistar rats (*n* = 60) weighing approximately 200 ± 10 g were obtained from the center for experimental animals, College of Pharmacy, King Saud University, Riyadh. The rats were transported in specialized cages and brought to the animal house, Department of food science and nutrition, College of food and agriculture sciences, King Saud University. The rats were maintained in standard environmental conditions, such as 25 ± 5ºC temperature, 50 ± 5% relative humidity, and 12/12 h of lighting/darkness cycle [14]. Animals were acclimatized to our own animal house conditions, fed with standard rat chow pellet (American Society for Nutrition guidelines) and free access to water. Streptozocin (STZ) was used to induce diabetes mellitus (DM), further the animals were divided in to two groups such as, non-diabetic (*n* = 30) and diabetic (*n* = 30) rats. All the biochemical techniques and analysis were in accordance with the National Institute of Health guidelines for the care of laboratory animals (NIH), Department of health service (pages, 83 − 23, revised in 1993) [15]. All the animal experiments were carried out according to the institutional ethical norms approved by the ethics committee, College of Medicine, King Saud University [IRB no. KSU-SE-19-124].

### Experimental induction of STZ-induced diabetes

After initial determination of 12 h fasting blood glucose levels, induction of experimental type 2 diabetes was achieved by intraperitoneal administration of STZ with a minimal dose of 45 mg/kg dissolved in 0.1 M citrate buffer (pH 4.5), freshly prepared [16]. The control animals were injected with citrate buffer (pH – 4.5) alone. After 8 h of STZ administration, the rats were allowed to intake 15% glucose solution through their dripping bottles attached with their cages to prevent hypoglycemia for next 24 h [17]. Diabetes was developed and stabilized in STZ injected rats within the period of 7 days. After 7 days, the plasma glucose levels of each rat were determined, the rats identified with fasting plasma glucose (FPG) range of 280–350 mg/dl were considered diabetic and included in the study [16]. Blood was collected by sinocular puncture. Body weights, water intake, and fasting plasma glucose levels were measured at weekly intervals, whereas other parameters were assessed at the start and end of the experiment.

### Dietary lipid composition

The standard pellet diet consists of raw fat, crude protein, raw fat, raw fiber, cinders, salt, calcium, phosphorus, vitamin A, vitamin D and vitamin E were provided to control group. Overall the percentage of fat in standard pellet diet is 4%. The experimental healthy and diabetic groups supplemented with 15% increased amount of fat by adding the experimental dietary lipids (oils), the total fat have been determined as 19% in the supplementation diet (Table 1). The amount of fat in the standard diet was modified by pulverizing it and adding 150 g of different type of fat per kg of standard diet. To avoid the oxidation of fats or modifying their properties, the diet was prepared freshly every week. Fresh Al-Naimi’s sheep fat (a form of saturated fat) acquired from a local market, the sheep’s fat was heated in a water bath (upto 70 °C), to transform it from solid to liquid. Coconut oil prepared based on its translucent cold mechanical squeeze upon melting from the land of nature industry is organic virgin coconut oil, supplied from Ceylon Naturals, Srilanka. The olive oil (extra virgin, cold-pressed) was purchased from the local market, supplied by the Alwazer Company, Spain. The corn oil (Afia International Company) was purchased from local hypermarkets as a sort of dietary vegetable fat supplied by Savola Foods, Saudi Arabia. Fatty acid compositon in sheep fat, coconut oil, olive oil and corn oil were detrmined using GC-MS (Agilent 7890 A, Agilent Technologies, USA). NIST libraries were used to identify and interpret GC-MS data. The chemical name and concentration of each compound was expressed based on peak area percentage were presented in Table 2.

**Table 1:** Components of experimental diet (g/100 g)

	Components				
Nutrients (%)	Control Group Standard Food	Sheep Fat Group	Coconut Oil Group	Olive Oil Group	Corn Oil Group
Crude Protein	20%	20%	20%	20%	20%
Raw fat	4%	15%	15%	15%	15%
Raw fiber	3.50%	3.50%	3.50%	3.50%	3.50%
cinders	6%	6%	6%	6%	6%
Salt	0.50%	0.50%	0.50%	0.50%	0.50%
Calcium	1%	1%	1%	1%	1%
Phosphorus	0.60%	0.60%	0.60%	0.60%	0.60%
Vitamin A	20 IU/kg	20 IU/kg	20 IU/kg	20 IU/kg	20 IU/kg
Vitamin D	2.20 IU/kg	2.20 IU/kg	2.20 IU/kg	2.20 IU/kg	2.20 IU/kg
Vitamin E	70 IU/kg	70 IU/kg	70 IU/kg	70 IU/kg	70 IU/kg

**Table 2:** Compositon analysis in sheep fat, coconut oil, olive oil and corn oil using GC-MS analysis

S. No	Compound Name	Peak area (%)			
		Sheep fat	Coconut oil	Olive oil	Corn oil
1.	Nitroso-o-Ethylhydroxylamine	-	-	0.14	0.15
2.	Methylester pentadecanoic acid	-	-	0.23	-
3.	Butanoic acid	-	0.33	-	0.05
4.	2-Pyrrolidinone	-	0.46	-	-
5.	2-Deoxy chloroacetic acid	-	2.46	-	-
6.	1-octadecane	-	0.22	-	-
7.	Hexadecenoic acid	-	2.08	0.82	2.30
8.	Pentadecanoic acid	-		8.86	13.06
9.	Propanoic acid	-	0.30	-	-
10.	9,12-Octadecadienoic acid (ω-6)	-	-	8.73	51.34
11.	9-octadecanoic acid (Oleic acid)	29.28	5.5	54.12	23.58
12.	6- octadecanoic acid	-	-	0.36	1.20
13.	Vanadium	-	-	0.46	
14.	Cis-13,16-Docasadienoic acid	-	-	-	0.38
15.	Hexamethyl-cyclotrisiloxane	-	-	1.29	0.13
16.	Cyclotrisiloxane	-	-	0.58	0.10
17.	Beta-tocopherol	-	-	-	0.50
18.	4-methyl-2-trimethylsiliyloxy-acetophenone	-	-	-	1.08
19.	Squalene	-	-	15.70	-
20.	Capric acid	0.35	7.6	-	-
21.	Lauric acid	0.52	48.9	-	-
22.	Myristic acid	7.89	18.6	-	-
23.	Palmitic acid	28.58	13.7	-	-
24.	Palmitoleic acid	0.71	-	-	-
25.	Stearic acid	29.40	3.1	-	-
26.	Arachidonic acid	-	1.9	-	-
27.	Pentadecylic acid	0.81	-	-	-
28.	Margaric acid	1.98	-	-	-
29.	Linoleic acid	1.72	-	-	-

### Diet preparation and intervention with different dietary lipid

The rats comprising in a healthy group, were randomly allocated into five separate groups (*n* = 6). Group-1 (control group) was provided with a typical control diet that consisted solely of 4% maize oil as the primary source of dietary fat. Group-2 was provided with a standard diet enriched with sheep fat, resulting in a fat content of 15%. Group-3 received a standard diet enriched with 15% coconut oil. Group-4 was administered with a standard diet enriched with 15% olive oil. Finally, Group-5 was given a standard diet enriched with 15% addition of corn oil. The diabetic rats were subsequently assigned at random to five groups (*n* = 6), consisting of a control (group-6), a sheep fat (group- 7), a coconut oil (group-8), an olive oil (group-9) and a corn oil (group-10). All the groups in healthy and diabetic rat were subjected to the same condition and treatment as outlined in Table 3.

**Table 3:** Dietary lipids type used in feeding diabetic and non-diabetic rats’ diets

	Group I / rat	Group II/ rat	Group III / rat	Group IV / rat	Group V / rat
Healthy	5% corn oil	15% sheep fat	15% olive oil	15% coconut oil	15% corn oil
Diabetic	5% corn oil	15% sheep fat	15% olive oil	15% coconut oil	15% corn oil

The dietary lipids supplementation was given for the period of 45 days. No noticeable irritation or restlessness was observed throughout the experimental period. At the end of the 45th day, all the rats were sacrificed by decapitation under phenobarbitone sodium anesthesia (60 mg/kg) according to the ethical committee guidelines. Blood was collected in two different tubes; one was used for serum separation (lipid analysis), and the other was supplemented with an anticoagulant (sodium fluoride) for plasma glucose, insulin and protein assay. Liver tissues were collected, washed in ice-cold saline, and weighed.

### Calculation of earned weight and consumed food

Basis of the amount of food consumed throughout the 45-day trial period, the weight gained was calculated according to the following formula: *Weight Gained =* End of trial weight – Trial beginning weight [18]. The amount of food consumption was calculated using the equation: *Amount of food consumed (g) =* weight of food provided daily - weight of food lost per day × trial duration 45 days [18]. In addition, the food consumption efficiency was also calculated by the equation: *Food Consumption Efficiency =* Weight Gained (g) ÷ Food Consumed (g) [18].

### Fasting plasma glucose and lipid profile analysis

After blood sampling, plasma and serum were collected after centrifugation for all the analysis. Fasting plasma glucose was estimated colorimetrically using commercial diagnostic GOD-POD kit (Sigma Chemical Co., St. Louis, MO, USA) method [18]. Plasma insulin level (mIU/ml) was quantified using rat insulin enzyme linked immunosorbent assay (ELISA) kits (RayBiotech Inc., Norcross, GA, United States). Lipid profile was performed using blood chemistry analyzer (Response® 910, Diagnostic System-SIEMENS, Munich, Germany) for the following parameters; low density lipoproteins (LDL), high density lipoproteins (HDL) and total protein in plasma. The serum triglycerides (TG) and total cholesterol (TC) levels after the dietary intervention were analyzed [19]. The activity of plasma lipoprotein lipase (LPL) was analyzed using STA-610, LPL standard and activity assay kit, Cell Biolabs, INC, San Diego, USA.

### Analysis of oxidative stress markers

The level of lipid peroxide (LPO), glutathione (GSH) (Ransel kit, Randox Laboratories, Ltd., Crumlin, UK); the activity of catalase (CAT), superoxide dismutase (SOD) (Cayman, Rockford, USA) and glutathione peroxidase (GPx) (Randox Company, Antrim, UK) were quantified in both normal and diabetic rats using kit method. The total protein was quantified using assay kit (Bio-Rad, USA).

### Analysis of lipid metabolism regulators in liver

Liver tissues was collected, and 0.3–0.5 g was homogenized with 1:10 ratio of 50 mM Tris/HCl buffer (pH 7.5) in cold temperature. The homogenate solution was collected and centrifuged at 20,000 g for 15 min at 4ºC. The supernatant was collected to analyze the metabolic and inflammatory markers assays. High dietary fatty acid associated glucose-lipid oxidative metabolism regulating protein, like Fibroblast growth factor-21 (FGF-21), Melanocyte-stimulating hormone (MSH), Atp-binding cassette transporter-1 (ABCA-1), Sterol regulatory element binding protein-1c (SREBP-1c), and Peroxisome proliferator activated receptor- gamma (PPAR-γ) and Peroxisome proliferator activated receptor- alpha (PPAR-α) levels were analyzed in both normal and diabetic rats using ELISA kit (Quantikine R&D Systems, MN, USA).

### Analysis of serum inflammatory markers

After 45 days supplementation of dietary different lipid, the metabolic syndrome associated inflammatory markers such as, Tissue necrotic factor- alha (TNF-α) and Interleukin − 6 (IL-6) (R & D Systems, Minneapolis, MN, USA); Monocyte chemoattractant protein- 1 (MCP-1), Interleukin-1 beta (IL-1β), and Toll like receptor- 4 (TLR-4) (Quantikine R&D Systems, MN, USA) concentrations were analyzed in both normal and diabetic rats using an enzyme-linked immunosorbent assay (ELISA) kit method, according to their protocol.

### Statistical analysis

The experimental data was processed using the Statistical Package for Social Sciences (SPSS) software, which employed the following statistical procedures. The arithmetic means (M) and standard deviations (SD) were calculated. The significance between the treatments between the normal and diabetic groups were demonstrated using one-way ANOVA analysis. The differences in p-values less than 0.05 and 0.001 were deemed statistically significant [20]. In the occurrence of statistically significant differences utilizing variance analysis, the LSD post-test was employed to conduct multiple comparisons between the means.

## Results

### Impact of supplementation with different types of lipids on nutritional parameters in the healthy and diabetic group

The equivalence of the mean body weights of rats in the healthy and diabetic groups at the beginning of the experiment was presented in Table 4. The average body weight ranged between 200 ± 10 g, and there was no statistically significant difference at the level of 0.05 between the average weights, which indicates their suitability for the experiment. At the end of the experiment, the data indicate the equivalence of body weights in healthy groups where ranged from 286.67 to 308.50 g, and there was no statistically significant difference between the groups. The value of weight gain from the highest to the lowest found in the groups, such as in sheep fat, 89.50 ± 13.85 g, corn oil 85.17 ± 8.40 g, coconut oil 83.67 ± 8.62 g, olive oil 80.50 ± 19.93 g, control group 73.17 ± 28.27 g when compared to the initial weight. There were no significant differences between healthy groups for the weight gained, food intake, food consumed and food efficiency.

**Table 4:** The effect of consuming different types of dietary lipids on the quantity of weight gained (g), food consumption (g), and food efficiency (g), in the healthy group of rats

Groups Measurements	Control	Sheep Fat	Coconut Oil	Olive Oil	Corn Oil
**Body Weight (gm)**					
Healthy Initial	198.17 ± 14.74	201.50 ± 18.78	203.67 ± 17.90	204.17 ± 10.46	202.00 ± 15.81
Final^¥^	286.67 ± 31.78	289.33 ± 16.78	308.50 ± 12.79*	302.33 ± 20.98*	297.50 ± 18.17
**Diabetic**					
Initial	205.60 ± 16.29	193.00 ± 11.42	207.83 ± 13.09	194.00 ± 4.52	203.50 ± 10.73
Final^¥^	201.00 ± 29.44	189.67 ± 44.89	235.17 ± 23.22*	226.83 ± 24.88*	237.00 ± 35.91*
**Weight Gained (gm)**					
Healthy	73.17 ± 28.27	89.50 ± 13.85	83.67 ± 8.62	80.50 ± 19.93	85.17 ± 8.40
Diabetic^¥^	28.20 ± 17.71	44.75 ± 15.25	48.20 ± 15.20**	38.60 ± 14.88**	43.50 ± 12.94
**Food intake (gm)**					
Healthy	23.29 ± 1.91	21.96 ± 2.05	21.31 ± 1.98	21.54 ± 2.99	20.37 ± 1.74
Diabetic^¥^	25.44 ± 0.71	21.40 ± 2.24	22.64 ± 3.34	23.55 ± 3.45	21.85 ± 3.75
**Total food consumed (gm)**					
Healthy	652.13 ± 53.52	614.85 ± 57.40	596.67 ± 55.48	603.11 ± 83.71	570.23 ± 48.84
Diabetic^¥^	686.97 ± 19.08	577.85 ± 60.50	611.37 ± 90.11	635.93 ± 93.18	590.02 ± 101.31
**Food efficiency (%)**					
Healthy	3.09 ± 0.99	4.10 ± 0.66	3.95 ± 0.49	3.73 ± 0.73	4.20 ± 0.52
Diabetic^¥^	1.11 ± 0.70	2.06 ± 0.62	2.18 ± 0.83	2.74 ± 2.03	2.01 ± 0.64

The values are presented as mean ± SD (*n* = 6). ^¥−^ significantly different when compared to healthy groups. ** *p* ≤ 0.001, significantly different when compared to other healthy or diabetic groups. * *p* ≤ 0.05, significantly different when compared to other healthy or diabetic groups

In diabetic groups, at the end of the experiment the average weights of diabetic rats were found as 200 ± 59 g. The highest weight gain was observed in coconut oil at 48.20 ± 15.20 g, sheep grease at 44.75 ± 15.25 g, corn oil at 43.50 ± 12.94 g, olive oil group at 38.60 ± 14.88 g, followed by the control diabetic group found with 28.20 ± 17.71 g. The coconut oil, olive oil and sheep fat supplementation were found to be significantly (*p* ≤ 0.05) increased in terms of food intake, food consumption, and food efficiency when compared to the control group and corn oil supplemented groups.

### Impact of supplementation with different types of lipids on changes in glucose (mg/100 ml), insulin levels (mIU/ml), serum LPL and lipid profile (mg/100 ml) in healthy and diabetic groups

Table 5 represents the changes in blood glucose and lipid profiles in healthy and diabetic rats after 45 days supplementation with different dietary lipids. We observed that there was no change in the blood glucose level between the groups. Notably, sheep fat supplementation alone shown mild increase in blood glucose levels (158.38 ± 43.50). In diabetic groups identified with a significantly increased level of blood glucose in sheep fat (476.78 ± 25.04) and coconut oil (558.97 ± 23.39) when compared to olive oil and corn oil groups. Plasma insulin levels in olive oil supplemented groups was significantly (*p* ≤ 0.001) higher (3 fold) when compared to diabetic control. Meanwhile, the insulin level was found to be significantly (*p* ≤ 0.05) high in olive oil and corn oil group when compared to sheep fat and coconut oil supplementation.

**Table 5:** The effect of consumption of various dietary lipids on glucose levels and lipid profile of healthy rat groups

Groups Measurements	Control	Sheep Fat	Coconut Oil	Olive Oil	Corn Oil
**Glucose level (mg/100 ml)**					
Healthy	124.89 ± 29.91	158.38 ± 43.50	143.20 ± 57.25	138.70 ± 16.58	121.62 ± 23.91
Diabetic^¥^	393.82 ± 28.78	476.78 ± 25.04**	558.97 ± 23.39**	360.16 ± 16.76**	322.50 ± 29.36**
**Plasma insulin levels (mIU/ml)**					
Healthy	1.6 ± 0.05	1.8 ± 0.08	1.6 ± 0.04	1.9 ± 0.07^¥^	1.5 ± 0.04
Diabetic^¥^	0.9 ± 0.01	1.4 ± 0.03**	1.9 ± 0.06**	2.7 ± 0.09**^¥^	2.3 ± 0.06**^¥^
**Serum Lipoprotein lipase (mUnits/mL)**					
Healthy	5.1 ± 0.02	3.9 ± 0.01	3.5 ± 0.01	4.8 ± 0.03^¥^	4.5 ± 0.02
Diabetic^¥^	1.6 ± 0.01	6.5 ± 0.02**	5.9 ± 0.03	9.2 ± 0.04**	7.3 ± 0.03**
**Triglycerides (mg/100 ml)**					
Healthy	38.45 ± 7.28	60.59 ± 30.51	45.38 ± 10.85	34.73 ± 13.85	37.83 ± 5.65
Diabetic^¥^	120.92 ± 6.90	263.26 ± 19.04**	236.77 ± 12.86**	107.05 ± 15.67^¥^	126.53 ± 16.56**^¥^
**Total cholesterol (mg/100 ml)**					
Healthy	63.58 ± 12.03	79.77 ± 15.50	89.88 ± 29.85**	74.04 ± 12.08	84.04 ± 20.83**
Diabetic^¥^	98.53 ± 9.19*	137.09 ± 14.11**	167.44 ± 16.64**	96.42 ± 11.19^¥^	93.34 ± 12.23^¥^
**HDL- C (mg/100 ml)**					
Healthy	48.15 ± 11.02	44.43 ± 10.51	44.19 ± 11.57	41.73 ± 6.04	47.80 ± 6.39
Diabetic	48.08 ± 8.50	45.34 ± 4.88**	66.13 ± 7.34^€,^ **	53.34 ± 6.58*	48.25 ± 5.24^¥^
**LDL- C (mg/100 ml)**					
Healthy	7.74 ± 3.49	23.22 ± 12.69**	36.81 ± 19.07*	22.77 ± 10.98**	28.67 ± 21.94**
Diabetic^¥^	26.26 ± 5.71	39.10 ± 8.70**	53.96 ± 9.11*	31.67 ± 6.87*	24.79 ± 4.69*^¥^

The values are presented as mean ± SD (*n* = 6). € *p* ≤ 0.05 significantly different when compared to healthy groups. ** *p* ≤ 0.001, significantly different when compared to other healthy or diabetic groups. * *p* ≤ 0.05, significantly different when compared to other healthy or diabetic groups. ^¥^*p* ≤ 0.05, significantly different when compared to sheep fat or coconut oil groups

Lipoprotein lipase (LPL) plays an essential role in lipid homeostasis especially after high fat diet intake, majorly by mediating the intravascular lipolysis of triglyceride rich lipoproteins. In the present study, healthy rats found with lower level of LPL in sheep fat and coconut oil supplemented rats compared to olive and corm oil supplementation. In diabetic rats, LPL level found to be significantly low in sheep fat and coconut oil supplementation. In healthy groups, control, corn oil and coconut oil supplementation found with higher level of LPL level when compared to sheep fat supplemented groups. In diabetic group, olive oil and corn oil supplemented found with a moderate high level of LPL when compared to control or healthy groups.

In healthy groups, sheep fat supplement found with a significantly (*p* ≤ 0.001) higher level of triglyceride (TG) level (mg/100 ml) when compared to all the other fatty supplementation. In diabetic group, the sheep fat (263.26 ± 19.04) and coconut oil (236.77 ± 12.86) fount to be significantly higher level of TG when compare to control (120.92 ± 6.90) and corn oil (126.53 ± 16.56) group. Most notably, supplementation of olive oil significantly (*p* ≤ 0.05) decreased (107.05 ± 15.67) the TG level in diabetic groups when compared to control diabetic groups.

Total cholesterol (mg/100 ml) level found to unaltered in healthy group supplemented with dietary lipids. In diabetic groups, coconut oil supplementation significantly (*p* ≤ 0.001) increased (167.44 ± 16.64) the total cholesterol level when compare to control, olive oil or corn oil supplemented diabetic rats.

The levels of high-density lipoproteins found to be unaltered in healthy groups at the end of 45 days after supplementation with different dietary lipids. In diabetic groups, HDL-c level significantly (*p* ≤ 0.05) increased in coconut oil groups when compared to all the other groups. LDL-c level have been increased three-fold in healthy groups supplemented with different dietary lipid groups when compare to control. In diabetic groups the corn oil supplementation significantly decreased the LDL-c level when compared to all the other supplementations. The coconut oil supplementation produced the highest level of LDL-c when compared to control or sheep fat supplementation.

Overall, coconut oil supplementation increased the HDL-c as well as LDL-c levels in diabetic rats when compared to all the other vegetable fat or animal fat supplementations. Corn oil supplementation significantly decreased the fasting blood glucose, triglyceride and LDL-c levels when compared to all the other vegetable fat or animal fat supplementation. Triglyceride and total cholesterol level have been significantly decreased in olive oil supplemented group when compared to another dietary lipid supplementation.

### Impact of supplementation with different types of lipids on oxidative stress and antioxidant parameters in healthy and diabetic rats

Table 6 showing the changes in plasma lipid peroxides and antioxidants levels in healthy and diabetic groups supplemented with different dietary lipid. In healthy rats, dietary lipid supplementation increased all the antioxidant parameters when compared to the control. In diabetes condition, the results found with a significantly (*p* ≤ 0.001) high level of LPO and lower levels of GSH, CAT, SOD and GPX activity in animals. Olive oil supplementation for 45 days, significantly (*p* ≤ 0.05) decreased LPO levels and an increased GSH, SOD, CAT and GPX levels in diabetic rats when compared to another dietary different lipid group.

**Table 6:** The effect of various dietary fats supplementation on oxidative stress and antioxidant parameters in healthy and diabetic rats

Groups Measurements	Control	Sheep Fat	Coconut Oil	Olive Oil	Corn Oil
**LPO**					
Healthy	0.06 ± 0.05	0.15 ± 0.09	0.13 ± 0.07	0.09 ± 0.04*	0.11 ± 0.03
Diabetic^¥^	0.28 ± 0.08	0.26 ± 0.07	0.23 ± 0.09	0.14 ± 0.06**	0.18 ± 0.08
**GSH**					
Healthy	4.1 ± 0.03	3.4 ± 0.3	3.5 ± 0.2	3.6 ± 0.09	3.7 ± 0.06
Diabetic^¥^	2.1 ± 0.3	2.2 ± 0.5	3.9 ± 0.08**	4.7 ± 0.06*^,¥^	4.9 ± 0.08*^,¥^
**SOD**					
Healthy	1.4 ± 0.02	1.6 ± 0.04	3.2 ± 0.02	2.4 ± 0.06	2.8 ± 0.07
Diabetic^¥^	3.6 ± 0.08	2.9 ± 0.07	6.3 ± 0.08**	5.3 ± 0.06**	5.9 ± 0.11**
**CAT**					
Healthy	1.9 ± 0.04	2.5 ± 0.03	4.1 ± 0.0.07	5.8 ± 0.09	5.2 ± 0.06
Diabetic^¥^	2.4 ± 0.03	2.7 ± 0.04	5.2 ± 0.09**	6.9 ± 0.04**^,¥^	5.8 ± 0.08**^,¥^
**GPX**					
Healthy	0.9 ± 0.01	1.2 ± 0.02	1.6 ± 0.04	2.5 ± 0.04	1.9 ± 0.01
Diabetic^¥^	3.4 ± 0.06	1.6 ± 0.01	3.9 ± 0.06**	4.6 ± 0.07**^,¥^	3.2 ± 0.04

[**Units**: LPO- expressed as 𝜇 moles of MDA formed/gram. GSH - One unit defined as the reduction of 1µmol/min GSSG. SOD activity- One unit corresponds to the quantity of the enzyme in 20µL of the sample solution that inhibits the reduction reaction with superoxide anion by 50%. CAT activity- the amount of the enzyme that can catalyze 1 µmol H_2_O_2_ within 1 min under the condition of pH-7. GPx activity- the activity was expressed as the conversion of 1 mM/min NADPH to NADP+]

The values are presented as mean ± SD (*n* = 6). ^¥−^ significantly different when compared to healthy groups. ** *p* ≤ 0.001, significantly different when compared to other healthy or diabetic groups. * *p* ≤ 0.05, significantly different when compared to other healthy or diabetic groups. ^¥^*p* ≤ 0.05, significantly different when compared to sheep fat or coconut oil groups

Corn oil and coconut oil supplementation also significantly (*p* ≤ 0.001) reduced level of LPO, increased activities of GSH, CAT, SOD and GPX when compared to the sheep fat and untreated control group. The antioxidant level found to be higher in diabetic group when compared to normal group in all the treatment group.

### Impact of supplementation with different types of lipids on changes in hepatic lipid homeostasis linked parameters in healthy and diabetic groups

**Fig. 1:**
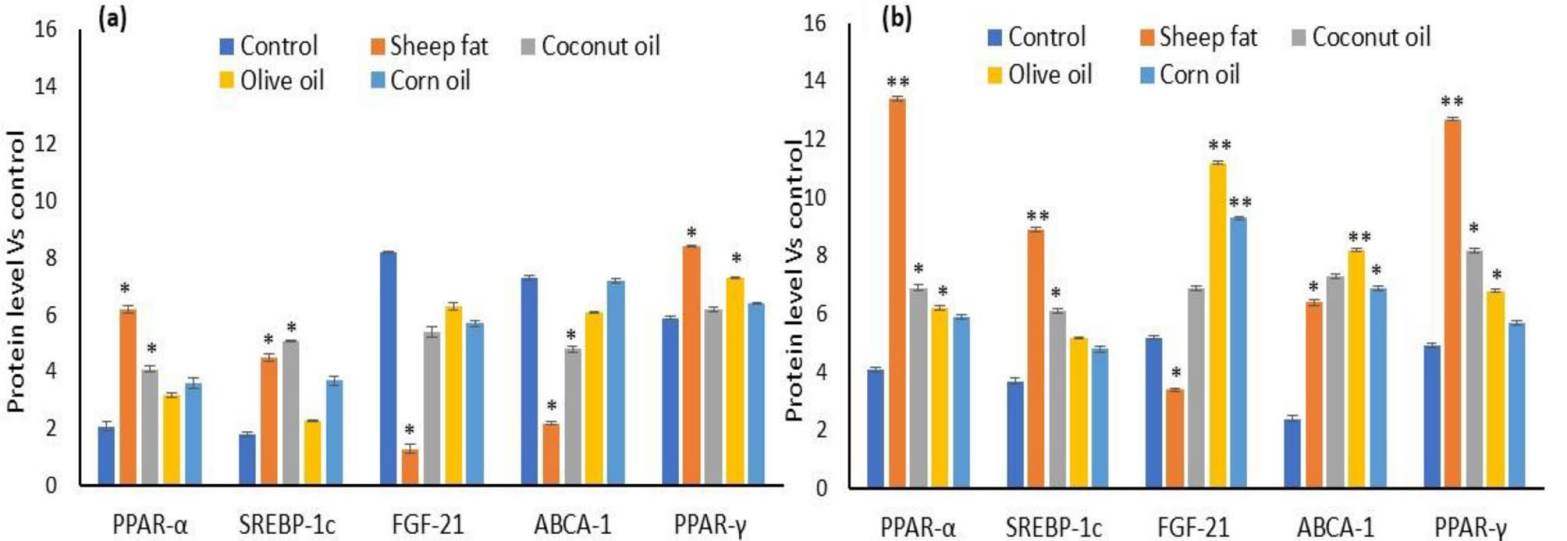
The effect of various dietary lipids supplementation on lipogenesis and glucose-lipid associated oxidative metabolism in healthy (**a**) and diabetic (**b**) rats.The values are presented as mean ± SD (*n* = 6). ** *p* ≤ 0.001, significantly different when compared to other healthy or diabetic groups. * *p* ≤ 0.05, significantly different when compared to other healthy or diabetic groups

**Fig. 2:**
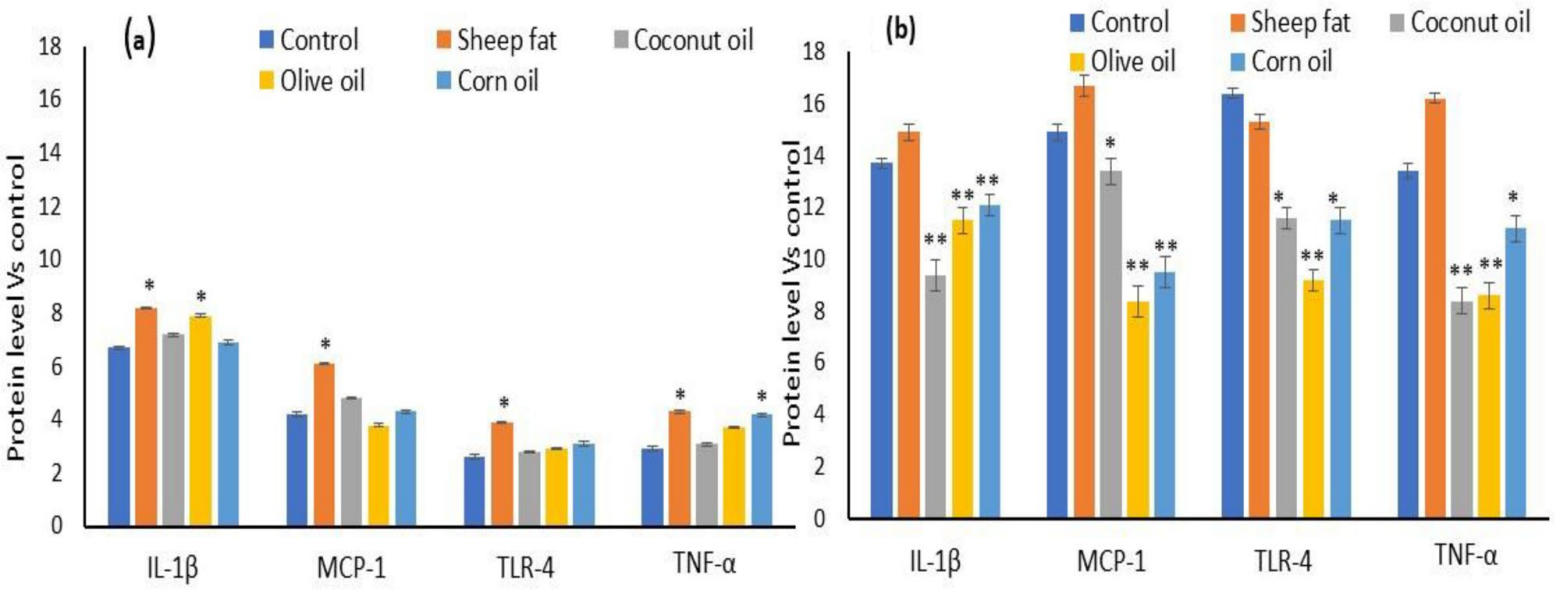
The effect of various dietary lipids supplementation on metabolic syndrome associated inflammatory markers in healthy (**a**) and diabetic (**b**) rats. The values are presented as mean ± SD (*n* = 6). ** *p* ≤ 0.001, significantly different when compared to other healthy or diabetic groups. * *p* ≤ 0.05, significantly different when compared to other healthy or diabetic groups

In the present study, control healthy rats found with lower level of PPAR-α, SREBP-1c and increased FGF-21 and ABCA-1 at the end of the experiment was presented in Figs. 1 and 2. Normal rats supplemented with sheep fat and coconut oil significantly increased lipogenesis associated proteins such as, PPAR- α, SREBP-1c, PPAR-γ and decreased FGF-21, ABCA-1 levels when compared to corn and olive oil supplemented rats. In healthy groups, corn oil and coconut oil supplementation found with higher level of glucose uptake, insulin sensitivity and cellular oxidative metabolism regulators such as, FGF-21, ABCA-1 and PPAR-γ level when compared to untreated control or sheep fat supplemented groups. In diabetic group, olive oil and corn oil supplemented found with a high level of FGF-21, ABCA-1 and PPAR-γ when compared to diabetic control or normal control groups. In addition, diabetic rats supplemented with olive oil and corn oil significantly increased levels of FGF-21, PPAR-γ, ABCA-1 and decreased levels of PPAR- α, SREBP-1c levels when compared to diabetic control, sheep fat and coconut oil treated groups. In diabetic rats, coconut oil supplementation significantly increased FGF-21, PPAR-γ and ABCA-1 level when compared to coconut oil supplemented healthy groups.

### Impact of supplementation with different types of lipids on changes in proinflammatory marker in healthy and diabetic groups

Metabolic syndrome associated inflammatory markers such as, IL-1β, MCP-1, TLR-4 and TNF-α were quantified in dietary lipid supplemented healthy and diabetic groups. In normal group, no change in the inflammatory markers such as, IL-1β, MCP-1, TLR-4 and TNF-α in between the different dietary lipid supplemented groups. Overall, olive oil and coconut oil group shown lower level of MCP-1, TLR-4 and TNF-α (not significant) in healthy group. Sheep fat supplemented healthy group shown moderate high in IL-1β and MCP-1 levels. In diabetic group, olive oil and corn oil supplemented group found with decreased level of IL-1β, MCP-1, TLR-4 and TNF-α when compared to diabetic control or sheep fat supplemented group. Diabetic rats supplemented with coconut oil found with no changes in the levels of IL-1β, MCP-1 and TNF-α, confirmed the lower risk of metabolic inflammation. In addition, diabetic rats supplemented with sheep fat found with a significantly increased levels of IL-1β, MCP-1 and TNF-α when compared to coconut oil treated groups.

## Discussion

Chronic excessive nutrient intake, like the consumption of a high-fat diet (HFD), is increasingly recognized as a causative factor contributing to pro-inflammation, T2D and specific cancers [21]. However, not all fats contribute to metabolic inflammation, for example saturated fatty acids (SFAs) are implicated in metabolic inflammation. Whereas the unsaturated fatty acids such as monounsaturated fatty acids and polyunsaturated fatty acids, have ameliorate metabolic inflammation and maintain energy balance [22]. In the present study, we compared the dietary supplementation of animal fat (sheep fat) against the vegetable fats rich in saturated fat (coconut) as well as high amount of poly and monosaturated fatty acids rich (olive and corn oil) on metabolic inflammation and progression of secondary complication. Sheep meat consist of 55% of saturated fat and remaining portion covers the unsaturated, monosaturated, n-6 and n-3 fatty acids [23] but the sheep grease consists 98% of saturated fat [24]. Meantime, vegetable oils like coconut oil possess 90% of medium chain saturated fats (like, lauric acid), which differ from animal saturated fats; they are easily absorbed in small intestine and do not undergo degradation or re-esterification [25]. Olive oil and corn oil possess a lipid source respectively of monounsaturated and polyunsaturated fatty acids [26].

Supplementation of sheep fat (grease) increased the TG and total cholesterol level in normal as well as in diabetic rats, might be the composed of high saturated fat. Olive oil supplementation showing the increased LPL level significantly correlate with the lower level of TG in circulation in diabetic animals. In this context, increased LPL activity triggers the hydrolysis of TG -rich lipoprotein, releasing non-esterified fatty acid, which further utilized by the liver and peripheral tissues for energy production [27]. The reduced weight gain in olive oil or corn oil supplementation correlated to increased LPL activity, as compared with another lipid supplementation. The above findings were evidenced by the increased insulin level and decreased weight gain in olive oil and corn oil supplemented diabetic rats. Conversely, diet rich in saturated fats, such as sheep fat or coconut fat hinder the insulin response to LPL activity in adipose tissue. This disruption leads to increased adiposity due to the accelerated rate of TG storage, ends with insulin resistance and obesity [28]. Coconut oil supplementation increased both HDL and LDL-c levels in both healthy and diabetic groups. In addition, olive oil and corn oil supplementation effectively suppressed the inflammatory cytokines responsible for high fat diet induced obesity-associated insulin resistance and atherosclerosis. In this context, current dietary guidelines for addressing oxidative stress, metabolic inflammation and preventing T2D promote the awareness on reducing overall fat intake, particularly animal fats, coupled with an emphasis on increased consumption of vegetable fats [29, 30].

Excessive dietary fatty acid intake stimulates the lipogenesis signaling proteins such as, PPAR-α, SREBP-1c involves in triglyceride synthesis and insulin resistance by decreasing glucose homeostasis [10]. The glucose-lipid metabolism linked cellular thermogenesis regulator FGF-21 found to be decreased in sheep fat supplemented healthy and diabetic groups. In this study, we found an increased FGF-21 level in both normal and diabetic groups treated with vegetable fats, might be due to the increased insulin levels and LPL activity. FGF-21 increases insulin mediated glucose uptake in adipose tissue [31]. FGF-21 also inhibit lipolysis through suppressing lipase activity and lipid droplet linked perilipin [32]. In this context, Holland et al. [33] found that FGF21 stimulates adiponectin secretion in mice, which regulate insulin sensitivity and energy expenditure. In addition, we found a decreased weight gain and increased LPL activity in olive oil and corn oil groups, might be due to the stimulation of insulin sensitivity and energy expenditure. Short chain fatty acid and monounsaturated fatty acid containing, coconut oil, olive oil and corn oil increased FGF-21 expression; it may be due to the lipid absorption and stimulation of oxidative energy production. The above findings were correlates with the higher weight gain found in sheep fat and coconut oil, confirmed the high lipogenesis than fatty acid oxidation. In this context, Staiger et al. [34] have found that FGF-21 stimulates the oxidation of fatty acids, the production of ketone bodies, and the inhibition of lipogenesis. Many researches evidenced that FGF-21 regulates glucose-lipid metabolism has made it a promising therapeutic target for metabolic disease [35, 36].

ATP-binding cassette transporter-1 (ABCA-1) mediates the transport of cholesterol and phospholipids from cells to HDL apolipoproteins, which increase HDL levels and inhibit atherogenesis [37]. Saturated fatty acid supplementation develops abnormal high-density lipoprotein (HDL) metabolism in diabetic condition and insulin resistance may contribute to their increased risk of atherosclerosis. In healthy groups supplementation of short chain fatty acids, MUFA containing coconut oil, olive oil and corn oil increased ABCA-1 and HDL levels. Meanwhile in diabetic condition, the ABCA-1 and HDL levels decreased in high fatty supplementation, especially in sheep fat and coconut oil. Dietary or metabolic factors which modulate ABCA1 activity might possess a profound lead on cholesterol transport and atherosclerosis [38]. Dietary fats encompass a diverse array of fatty acids with many biological functions, exerting a significant influence on metabolic pathways that affect the risk of metabolic syndrome, especially metabolic inflammation, T2D and cardiovascular problems [39].

Nuclear receptors, especially peroxisome proliferator-activated receptor (PPAR-α and PPAR-γ) are ligand-activated transcription factors that regulate the metabolism of glucose and lipids homeostasis [40]. Monounsaturated fatty acid (MUFA) and short-chain fatty acid (SCFA) supplementation effectively regulate the lipid clearance and cellular fatty acid oxidation stimulators like, PPAR-α and PPAR-γ in healthy and diabetic animals [41]. Control diabetic groups and saturated animal fat fed groups considerably raised (*p* ≤ 0.05) the PPAR-α, SREBP-1c levels compared to the healthy control group, meanwhile the vegetable fat fed groups were found with unaffected PPAR-α and increased PPAR-γ levels.

The development of persistent systemic inflammation induced by high-fat diets (HFD) activates the Toll-like receptor (TLR) signaling pathway, resulting in increased permeability of the intestines to endotoxins such as lipopolysaccharides (LPS) [42]. Furthermore, elevated levels of free fatty acids (FFAs) found in HFDs may directly impact immune system, especially on macrophage cells [43]. Increased plasma FFAs and oxidative stress stimulate TLRs in circulating macrophages, causing their activation into a pro-inflammatory M1 phenotype, which subsequently produces inflammatory cytokines such as interleukin (IL)-1β, MCP-1, and tumor necrosis factor (TNF)-α associated with metabolic inflammation [44, 45]. Toll like receptor (TLR-4) and monocyte chemoattractant protein-1 (MCP-1) have been reported to be a novel adipocytokine involved in the development of obesity-associated insulin resistance and atherosclerosis [42].

Elevated oxidative stress has been found mainly in diabetic conditions, due to an increased production of oxygen free radicals from excessive dietary lipid source, which cause reduction of antioxidant defenses [46, 47]. The endogenous antioxidant enzymes (e.g. SOD, CAT, GSH and GPx) are the defense system which detoxify the oxygen radicals [48]. Reduced activities of these antioxidant enzyme in liver, kidney and pancreas tissues have been observed in diabetic rats and this activity may result in a number of deleterious effects due to accumulation of superoxide anion (O) and hydrogen peroxide (H_2_O_2_), which in turn generate hydroxyl radicals (OH), resulting in initiation and propagation of LPO [46]. Decrease in the antioxidant status might be due to the excessive free radicals generation as well as excessive glycation to antioxidant proteins, which increase the amount free radicals [49]. Present study confirmed that the diabetic control and saturated fat group animals found with an increased lipid peroxidation and low in antioxidant (GPx, SOD and catalase) levels represents high oxidative stress. Noteworthy that the elevation of the GSH levels and antioxidant enzymes in vegetable lipid diet supplementation, which protects the cell membrane against oxidative damage by regulating the redox status of protein in the membrane. Overall control of lipid peroxidation and cellular oxidation by MUFA and SCFA rich olive oil and corn oil supplementation effectively control the metabolic syndrome associated lipid peroxides and inflammatory cytokine progression. Olive oil and corn oil supplementation might increase insulin mediated cellular uptake of free fatty acids through FGF-21, further SREBP-1c stimulate mitochondrial oxidation and energy production. Moreover, dietary recommendations suggest increased consumption of monounsaturated fatty acids [50], polyunsaturated fatty acids [51], and omega-3 fatty acids [52], along with reduced intake of saturated fatty acids [29] and trans fatty acids [52] ameliorate metabolic lipid peroxides generation, oxidative stress and inflammation. Although meta-analyses have indicated that the prevention of insulin resistance, T2D incidence have been associated with vegetable fat intake and, the individual consumption of specific fatty acids, such as saturated fatty acids, monounsaturated fatty acids, and polyunsaturated fatty acids, did not demonstrate a significant association with the incidence of T2D [51, 53].

## Conclusion

Healthy animals supplemented with saturated fat stimulated the lipogenesis pathway and suppressed the lipolytic signaling proteins, which resulted in high levels total cholesterol and triglyceride levels. Meantime, SCFA or MUFA supplementation to health groups significantly stimulated the FGF-21, SREBP-1c and ABCA-1 expression, aid the oxidation of fatty acids, and the inhibition of lipogenesis was confirmed by the low levels of TG and inflammatory cytokines. In diabetic condition, supplementation of saturated fat or animal fat increased metabolic syndrome linked inflammatory markers could be resulted in progression of insulin resistance or atherosclerosis. Most notably, SCFA or MUFA rich coconut oil, olive oil and corn oil supplementation effectively reduced the inflammatory markers followed by the stimulation of lipogenesis and fatty acid oxidation signaling proteins. This effect might arrest the progression of metabolic inflammation or secondary complication.

## Data Availability

The datasets and analyses generated during this study are available with the corresponding author upon reasonable request. The data are not publicly available due to privacy.

## Abbreviations

ABCA-1: ATP-Binding Cassette Transporter-1
BW: Body Weight
CAT: Catalase
ELISA: Enzyme Linked Immunosorbent Assay
FGF-21: Fibroblast Growth Factor-21
GOD-POD: Glucose Oxidase –Peroxidase
GPX: Glutathione Peroxidase
GSH: Glutathione
HDL: High Density Lipoproteins- Cholesterol
IL-1β: Interleukin-1 Beta
LDL-C: Low Density Lipoproteins- Cholesterol
LPL: Lipoprotein Lipase
LPO: Lipid Peroxide
MCP-1: Monocyte Chemoattractant Protein- 1
MSH: Melanocyte-Stimulating Hormone
MUFA: Monounsaturated Fatty Acid
NIH: National Institute Of Health
PPAR-α: Peroxisome Proliferator Activated Receptor- Alpha
PPAR-γ: Peroxisome Proliferator Activated Receptor- gamma
SCFA: Short-Chain Fatty Acid
SFA: Saturated Fatty Acids
SOD: Superoxide Dismutase
SREBP-1c: Sterol Regulatory Element Binding Protein-1c
STZ: Streptozotocin
T2D: Type 2 Diabetes Mellitus
TFA: Trans Fatty Acids
TLR-4: Toll Like Receptor- 4
TNF-α: Tissue Necrotic Factor- Alpha
